# Molecular detection of *Anaplasma phagocytophilum* and *Borrelia burgdorferi* sensu lato genospecies in red foxes (*Vulpes vulpes*) from Romania

**DOI:** 10.1186/s13071-015-1130-9

**Published:** 2015-10-08

**Authors:** Mirabela Oana Dumitrache, Ioana Adriana Matei, Angela Monica Ionică, Zsuzsa Kalmár, Gianluca D’Amico, Sándor Sikó-Barabási, Dan Traian Ionescu, Călin Mircea Gherman, Andrei Daniel Mihalca

**Affiliations:** Department of Parasitology and Parasitic Diseases, University of Agricultural Sciences and Veterinary Medicine Cluj-Napoca, Faculty of Veterinary Medicine, Cluj-Napoca, Romania; Department of Ecological and Biotechnological Systems, Faculty of Environmental Science Babeş – Bolyai University, 14 Stadionului Street, 520064 Sfântu Gheorghe, Romania; Department of Game and Wildlife, Faculty of Silviculture and Forestry Engineering, Transilvania University, Şirul Beethoven 1, 500123 Braşov, Romania

**Keywords:** Red foxes, *Anaplasma phagocytophilum*, *Borrelia burgdorferi*, Tick-borne pathogens, Romania

## Abstract

**Background:**

Red foxes (*Vulpes vulpes*) are one of the most widespread wild carnivores in the world, being recognized to harbor and transmit a wide range of vector-borne diseases. *Anaplasma phagocytophilum* and *Borrelia burgdorferi* sensu lato are zoonotic tick-borne pathogens causing emerging diseases. Wild animals play an essential role in the transmission of diseases and pathogens maintenance in nature. Epidemiological studies regarding the prevalence of tick-borne pathogens in red foxes are of public health importance, as they may successfully act as a pathogen transmission interface between wildlife, domestic animals and humans.

**Findings:**

This study included 14 counties from Romania. A total number of 353 red foxes (*Vulpes vulpes*) were examined. Heart tissue samples were collected during necropsy and stored at −20 °C. Genomic DNA extraction was performed and all samples were examined by polymerase chain reaction (PCR). Specific primers for *A. phagocytophilum*, *A. platys*, *E. canis* and *Borrelia burgdorferi* s.l. were used. Sequence analysis was performed (Macrogen Europe, Amsterdam) and obtained sequences are available at GenBank™. Out of the 353 samples, 9 (2.55 %; 95 % CI: 1.25–4.96 %) were positive for *A. phagocytophilum*. Positive animals originated from 5 counties. In total, 5 out of 353 heart tissue samples (1.42 %; 95 % CI: 0.52–3.47 %) collected from red foxes were positive for *B. burgdorferi* s.l. Red foxes originated from 4 counties. None of the samples were positive for *A. platys* or *E. canis*. No co-infection with *A. phagocytophilum* and *B. burgdorferi* s.l. was found.

**Conclusion:**

This first report of *A. phagocytophilum* and *B. burgdorferi* s.l. in red foxes from Romania suggests a limited role of foxes in the maintenance of the two related pathogens, but may represent a potential risk from a public health perspective.

## Findings

### Background

Red foxes are one of the most widespread wild carnivores in the world and one of the most adapted species to synanthropic ecosystems [1]. Foxes play an important role in the ecoepidemiology of several tick-borne pathogens, serving as reservoir hosts for zoonotic agents such *Borrelia burgdorferi* [2] or as hosts for vectors [3] and contribute to disease dissemination to humans and domestic animals. Among these, *Anaplasma phagocytophilum* (the agent of human granulocytic anaplasmosis, canine and equine anaplasmosis and tick-borne fever in ruminants) and *Borrelia burgdorferi* s.l. (the agent of Lyme borreliosis) are both posing a real threat to public health. Due to increased awareness of medical personnel and improved diagnostic techniques, co-infections with more than one tick-borne pathogen are more commonly diagnosed in humans, animals and ticks. *A. phagocytophilum* and *B. burgdorferi* s.l. association has a particular clinical importance because co-infection intensifies the pathogenic process and increases the severity of the Lyme borreliosis as shown in animals [4]. Moreover, in Europe, the main vector for both pathogens is the same tick, *Ixodes ricinus* [5]. This species is widely distributed and has a low host-specificity being the dominant tick species in Romania [6].

Both transmission and maintenance of these pathogens in nature are following an enzootic lifecycle that involves ticks and a broad range of reservoir hosts [5] in which wildlife is an essential component [4]. Among wild canids, foxes are playing a key role, as they may act as an interface for pathogen transmission between wildlife, domestic animals and humans [1]. Despite this, little information is available on the occurrence of *A. phagocytophilum* and *B. burgdorferi* s.l. in red foxes in Europe.

The aim of this study was to detect the presence of *A. phagocytophilum* and *B. burgdorferi* s.l. in tissues of red foxes in Romania.

### Methods

#### Sample collection

**Fig. 1:**
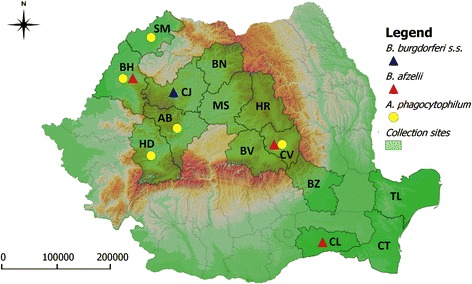
Collection sites and geographical distribution of red fox positive samples for *B. burgdorferi* s.s., *B. afzelii* and *A. phagocytophilum*. AB = Alba; BH = Bihor; BN = Bistriţa Năsăud; BV = Brașov; BZ = Buzău; CJ = Cluj; CL = Călărași; CT = Constanţa; CV = Covasna; HD = Hunedoara; HR = Harghita; MS = Mureș; SM = Satu Mare; TL = Tulcea

A total of 353 red foxes (*Vulpes vulpes*) from 14 Romanian counties (Fig. 1) were examined between October 2011 and May 2015. The animals were collected by the National Sanitary Veterinary Authority during the rabies monitoring program. All the animals negative for rabies were transported to our laboratory according to the current laws on dead animals transport and zoonotic risks. Heart tissue samples were collected during necropsy and stored at −20 °C until further examination.

#### DNA extraction

Genomic DNA extraction was performed using ISOLATE II Genomic DNA Kit, Bioline, following the manufacturer’s instructions. For each extraction procedure, negative controls (reaction mixture without tissue sample) were used in order to identify possible cross-contamination. DNA from a representative number of samples was quantitatively evaluated using Nanodrop ND-1000 spectrophotometer analyser.

#### Polymerase chain reaction (PCR)

All DNA samples were screened by PCR. The primers EHR16SD (5′-GGT ACCYACAGAAGAAGTCC-3′) and EHR16SR (5′-TAGCACTCATCGTTTACAGC-3′) were used, and the *16S rRNA* gene (345-bp fragment) was amplified in order to detect *Anaplasma, Ehrlichia, Neorickettsia, Wolbachia* genera [7]. All positive samples were further examined using specific primers for *A. phagocytophilum*, *A. platys* and *E. canis*.

The presence of *A. phagocytophilum* was assessed by PCR using specific primers LA1/LA6 (forward primer: 5′-GAGAGATGCTTATGGTAAGAC-3′, reverse primer: 5′-CGTTCAGCCATCATTGTGAC-3′), amplifying a 444-bp fragment of *ankA* gene [8]. The DNA extracted from the blood of a dog naturally infected with *A. phagocytophilum* (provided by IDEXX Germany) was used as positive control. *A. platys* DNA was amplified using specific primers EPLAT5 (5′-TTTGTCGTAGCT TGCTATGAT-3′) and EPLAT3 (5′-CTTCTGTGGGTACCGTC-3′) targeting a 359-bp fragment of the 16S rRNA gene [9]. Specific *E. canis* amplification was performed using the following primers CANIS (5′-CAATTATTTATAGCCTCTGGCTATAGGA-3′), GA1UR (5′-GAGTTTGCCGGGACTTCT TCT-3′), targeting a 409-bp fragment of the 16S rRNA gene [10]. DNA extracted from blood samples of dogs naturally infected with *A. platys* and *E. canis* were used as positive controls (provided by Prof. Harrus). The detection of *B. burgdorferi* s.l. was performed targeting the region of 5S-23S rRNA (rrf-rrl) intergenic spacer (IGS) in Bio-Rad T1000 Thermal Cycler, according to a previously described protocol [11]. For each PCR reaction, positive (*B. burgdorferi* s.l. culture strains) were used as positive control. In all cases, a reaction mix without DNA was used as negative control.

The PCR reaction was carried out in a final volume of 25 μl using 2x Green DYE Master Mix (Rovalab GmBH). Amplicons were visualized by electrophoresis in a 1.5 % agarose gel (1 × TAE, pH 8.0) stained with SYBR® Safe DNA gel stain (Invitrogen).

#### Sequencing

For all *A. phagocytophilum* positive samples (targeting 16S rRNA gene) and For *B. burgdorferi* s.l. positive samples, PCR products were purified using QIAquick PCR Purification Kit (QIAGEN). Sequence analysis was performed (Macrogen Europe, Amsterdam) and the sequences were compared to those available in GenBank™ dataset by Basic Local Alignment Tool (BLAST) analysis.

Sequences were submitted to the GenBank™ under the following accession numbers: KT351866, KT351867 and KT751324.

#### Statistical analysis and mapping

Statistical analysis of the results, prevalence of pathogens, its 95 % confidence interval (95 % CI) and *p* value, were performed using the EpiInfo 2000 software (CDC, USA).

The map including collection sites and positive counties for *A. phagocytophilum* and *B. burgdorferi* genospecies was generated using QGIS software.

#### Ethics statement

All aspects of sample collections were carried out in the framework of the disease control activities implemented and approved by the Ministry of Health and Ministry of Agriculture and Rural Development and adopted by regional and local administrative and veterinary and health authorities.

### Results

Out of the 353 heart tissue samples collected from red foxes, 9 (2.55 %; 95 % CI: 1.25–4.96 %) were positive for DNA of Anaplasmataceae family. Specific amplification of all samples showed their positivity for *A. phagocytophilum*-specific DNA. All samples were negative for *A. platys* or *E. canis. A. phagocytophilum* positive animals originated from 5 counties (Alba, *n* = 1; Bihor, *n* = 2; Covasna, *n* = 3; Hunedoara, *n* = 1; Satu Mare, *n* = 2) (Fig. 1). No significant differences were observed between counties regarding the prevalence of *A. phagocytophilum*.

All sequences obtained from positive samples were identical to each other. The sequence was found to be 99 % identical to *A. phagocytophilum* strains.

Out of all samples, 5 (1.42 %; 95 % CI: 0.52–3.47 %) were positive for *B. burgdorferi* s.l. The sequenced *rrf-rrs* gene highlighted 98 % and 100 % similarities with *B. burgdorferi* sensu stricto (s.s.) and *B. afzelii* respectively.

Four samples (1.13 %; 95 % CI: 0.36–3.08 %) from Covasna (*n* = 2), Bihor (*n* = 1) and Călărași (*n* = 1) counties were infected with *B. afzelii*. One sample from Cluj (0.28 %; 95 % CI: 0.01–1.82 %) was infected with *B. burgdorferi* s.s. (Fig. 1).

No co-infections were found.

### Discussion

Red foxes are among the most widespread and abundant wild carnivores. Their adaptation to urban environment and human presence, their frequent exposure to tick bites and the reservoir or maintenance host role for humans and domestic animals pathogens, highlight their importance for public health. However, no data concerning the epidemiology of tick-borne diseases in red foxes in Romania are available. To our knowledge, this is the first study investigating tick-borne pathogens in tissue samples collected from red foxes in Romania.

In Europe the information about the prevalence of *A. phagocytophilum* in foxes is scarce: Recorded prevalence range from 16.6 % in Italy [12], 8.2 % in Germany [13], 4 % in Czech Republic [14] to 2.7 *%* in Poland [15]. No positive foxes were found in studies conducted in Austria [1] or Bosnia and Herzegovina [16].

Few data regarding the prevalence of *A. phagocytophilum* in *I. ricinus* collected from foxes are available. In a recent study, Dumitrache et al. [17] found a prevalence of 4.4 % of the pathogen in *I. ricinus* collected from red foxes in Romania. In a similar study conducted in Hungary, the prevalence was 1.3 % [18].

Few data are available regarding the prevalence of *B. burgdorferi* s.l. in red foxes in Europe. In Germany, 24 % of the skin samples from foxes were PCR positive after cultivation [19]. In another study from Germany the prevalence in skin samples was 7 % following skin biopsy analysis [20]. However, a review on European reservoir hosts for *B. burgdorferi* s.l. suggests that red fox may be considered as a potential reservoir [2].

In Romania, foxes harbor *I. ricinus, I. hexagonus*, *I. crenulatus* and *Dermacentor marginatus* [21]. In Europe, *I. ricinus* is considered the main vector for both *B. burgdorferi* s.l. and *A. phagocytophilum*. Moreover, vectorial role of *I. hexagonus* for *B. burgdorferi* was also demonstrated [22]. Although *A. phagocytophilum* has been detected in engorged *I. hexagonus*, the vector competence of this tick species needs to be further investigated [23].

Although no co-infections with *A. phagocytophilum* and *B. burgdorferi* were found, our results showed that both pathogens are present in red foxes from the same counties (Bihor and Covasna), creating suitable epidemiological backgrounds for co-transmission. The role of red foxes in the ecoepidemiology of Lyme disease has not been completely elucidated. Older publications suggest that red foxes may serve as reservoir hosts [19, 20]. In addition, Kahl et al. demonstrated the transmission of the pathogen from experimentally infected red foxes to *I. ricinus* ticks [24]. The information regarding the role of red foxes in the life cycle of *A. phagocytophilum* is similarly scarce [1].

### Conclusions

To the best of our knowledge, *A. phagocytophilum* and *B. burgdorferi* were detected for the first time in tissues samples collected from red foxes in Romania. Our results indicate that foxes may be involved in the ecoepidemiology of these two pathogens by maintaining the infection in the synanthropic environments, posing an important risk for public health.
